# Ferroptosis in early brain injury after subarachnoid hemorrhage: review of literature

**DOI:** 10.1186/s41016-024-00357-4

**Published:** 2024-02-13

**Authors:** Junlin Kang, Shilai Tian, Lei Zhang, Gang Yang

**Affiliations:** 1 The First Hospital of Lanzhou University, Lanzhou City, Gansu Province China; 2 Gansu Provincial Hospital, Lanzhou City, Gansu Province China

**Keywords:** Subarachnoid hemorrhage, Early brain injury, Ferroptosis, Intracranial aneurysm

## Abstract

Spontaneous subarachnoid hemorrhage (SAH), mainly caused by ruptured intracranial aneurysms, is a serious acute cerebrovascular disease. Early brain injury (EBI) is all brain injury occurring within 72 h after SAH, mainly including increased intracranial pressure, decreased cerebral blood flow, disruption of the blood-brain barrier, brain edema, oxidative stress, and neuroinflammation. It activates cell death pathways, leading to neuronal and glial cell death, and is significantly associated with poor prognosis. Ferroptosis is characterized by iron-dependent accumulation of lipid peroxides and is involved in the process of neuron and glial cell death in early brain injury. This paper reviews the research progress of ferroptosis in early brain injury after subarachnoid hemorrhage and provides new ideas for future research.

## Background

Spontaneous subarachnoid hemorrhage (SAH) accounts for about 5% of all strokes, 80% of which is caused by the rupture of intracranial aneurysms [1]. It can also be secondary to cerebral arteriovenous malformation, moyamoya disease, vasculitis, or amyloid angiopathy [2]. Compared with other types of stroke, SAH has a high mortality rate and has a greater impact on young patients [3]. The incidence of SAH in China is about 2/100,000 per year, of which 15% of the patients die when the aneurysm ruptures and the 30-day mortality rate is as high as 45% [4]. The surviving patients usually leave different degrees of neurological and cognitive impairment, and 50–66% of patients will be permanently disabled [5]. After bleeding, intracranial pressure can sharply increase in a short period of time, causing global cerebral ischemia and hypoxia, activating inflammation, oxidative stress, and other factors that can lead to blood-brain barrier (BBB) disruption, brain edema, and ultimately brain tissue damage and neurological dysfunction [6]. Aneurysmal rebleeding occurs in 7–23% of patients, which is the main predictor of poor prognosis in patients with aneurysmal subarachnoid hemorrhage (aSAH) [7]. The incidence of delayed cerebral ischemia (DCI) is about 30%, mainly 4–12 days after SAH. It is manifested by cerebral vasospasm, cerebral autonomic regulation dysfunction, microthrombosis, and cortical spreading depolarizations, which is one of the causes of disability and death of patients [8]. It is generally believed that rebleeding of aneurysms, cerebral vasospasm, and delayed cerebral ischemia are the main factors affecting the prognosis of patients with subarachnoid hemorrhage [9]. However, anti-angiospasm drugs have not significantly improved the prognosis of patients in clinical trials [10]. In recent years, the pathophysiological mechanism of early brain injury after SAH has become a research hotspot. Early brain injury (EBI) is the total brain injury that occurs within 72 h after the onset of SAH and is one of the main pathologies for patient death and poor prognosis [11]. EBI was first proposed by Kusaka et al. in 2004 [12]. It is an important pathological process after SAH, and its specific mechanism is still poorly understood. The current research mainly comes from animal experiments and lacks clinical data. Many pathways are involved in its pathophysiological process, including raised intracranial pressure, oxidative stress, neuroinflammation, blood-brain barrier disruption, cerebral edema, microthrombosis, cortical spreading depolarizations, cerebral autoregulation dysfunction, decreased cerebral blood flow, and neurodegeneration [10, 13]. These changes lead to secondary brain injury, which is clearly manifested as cerebral ischemia, edema, and microcirculation disorders, causing activation of cell apoptosis and necrosis pathways [14], leading to neuronal death [15]. EBI lays the foundation for the development of DCI, so it is speculated that both cerebral vasospasm and DCI are related to EBI [8]. EBI reflects early clinical manifestations and is an important predictor of prognosis.

Complications of subarachnoid hemorrhage include increased intracranial pressure, cerebral vasospasm, delayed cerebral ischemia, epilepsy, neurogenic pulmonary edema, and hydrocephalus [16]. Multimodal monitoring data showed that brain oxygen content decreased and lactate pyruvate ratio increased, supporting the role of early cerebral hypoperfusion in EBI [17]. Based on the whole brain apparent diffusion coefficient (ADC) on magnetic resonance imaging (MRI), studies have quantified the degree of early cytotoxic brain edema and vascular brain edema and found that the ADC value of SAH patients was significantly increased. Therefore, early brain edema may be an important mechanism of EBI [18]. Brain edema also plays an important role in EBI and is an independent predictor of mortality [19], edema can further aggravate cerebral hypoperfusion and cause repeated circulation of secondary brain injury and cell death [17]. Neuroinflammation is a reaction after brain injury and maybe another key factor of EBI [20]. The early degradation products of red blood cells in SAH can activate inflammatory responses and accelerate brain damage [21]. Microglia are activated and participate in the inflammatory process within a few minutes after SAH [22]. They can promote neutrophil and macrophage activation by releasing pro-inflammatory factors and oxidative metabolites, further exacerbating brain injury, leading to blood-brain barrier disruption, inflammatory response, and neuronal damage [23]. Cortical spreading depolarizations (CSD) are slow-moving, self-propagating waves of neural and glial depolarization that cause damage through energy depletion, excitotoxicity, and spreading ischemia [24]. Up to 80% of low-grade SAH patients exhibit cortical diffuse depolarization [25]. CSD can occur immediately or within 2 weeks after SAH [26], usually triggered by high potassium ions released by red blood cell degradation or cortical loss caused by bleeding [27]. Iron is released from hemoglobin and has been shown to be an important toxin that causes neuronal death [28]. SAH induced neuronal death involves many pathways, including apoptosis, necrosis, and autophagy [29]. Studies have shown that the content of free iron in neurons increases after SAH and confirmed ferroptosis in early brain injury after subarachnoid hemorrhage [30]. At present, there is no targeted treatment for early brain injury, mainly to control intracranial pressure and optimize cerebral perfusion pressure [31]. Several related studies have shown that EBI is an important factor affecting the prognosis of SAH patients and that early intervention can reduce neurological dysfunction [32, 33]. Therefore, improving our understanding of EBI-related pathogenic factors will help to find new interventions and improve the prognosis of patients.

## Potential mechanisms of early brain injury

### Elevated intracranial pressure, changes in cerebral microcirculation, and energy metabolism disorders

In the acute phase of SAH, bleeding leads to an increase in intracranial pressure and a decrease in cerebral perfusion pressure, resulting in a decrease in cerebral blood flow and global cerebral ischemia. Blood entering the subarachnoid space can also affect cerebrospinal fluid circulation, and the occurrence of hydrocephalus may increase intracranial pressure and decrease cerebral blood flow, exacerbating brain injury. Impairment of brain automatic regulation function may be the main factor leading to an early decrease in cerebral blood flow [34]. Due to factors such as microvascular spasm, microthrombus formation, impaired self-regulation function, and disruption of the blood-brain barrier, the recovery of cerebral blood flow takes a longer time [35]. Microvascular spasm often occurs in small arteries. Microscopic examination of blood vessels in SAH mice revealed that 70% of arterial vessels experienced acute contractions lasting up to 72 h, while venous vessels remained unchanged [36]. The experiment of rat SAH showed that the vasodilation activity of cerebral arterioles changed [37]. The ultrastructural examination of microvasculature 1 h after SAH showed partially collapsed capillaries, enlarged astrocyte foot processes and endothelial cell luminal processes [38]. The NO produced by endothelial NO synthase (eNOS) plays an important role in maintaining microvascular function [39]. After SAH, the concentration of NO decreases, and inhaling NO can reverse microvascular dysfunction, improve cerebral perfusion, and alleviate cerebral edema, thereby improving neurological function prognosis [40].

The main causes of platelet aggregation and microthrombus formation are arterial injury and active bleeding. In the rat SAH model, microvascular platelet aggregation was observed to occur 10 min after SAH, reaching its peak at 24 h and beginning to decrease at 48 h [36]. The damage caused by platelet aggregation may include mechanical obstruction and the release of serotonin, ADP, and platelet-derived growth factor, promoting microvascular spasm and leading to decreased cerebral perfusion. Neutrophil extracellular traps (NETs) are associated with early microthrombus formation after SAH [41]. The application of urokinase type plasminogen activator (u-PA) can reduce microthrombus formation and thus lower the mortality rate [42].

The energy metabolism of the brain mainly includes oxygen metabolism and glucose metabolism. The incidence of brain metabolic disorders and brain tissue hypoxia increases in patients with global brain edema on imaging on the first day after SAH [43]. In the study, positron emission tomography (PET) was used to observe a decrease in cerebral metabolic rate of oxygen (CMRO_2_) during the acute phase of SAH, which may be related to a decrease in cerebral blood flow caused by increased intracranial pressure [44]. PET can also be used to detect areas of abnormal glucose metabolism in the brain of SAH patients [45]. In clinical studies, it has been found that the glucose concentration in the brain tissue of most patients is significantly lower than that in serum glucose, indicating an increase in brain tissue glucose consumption [17]. The intake of glucose by astrocytes and pericytes is approximately four times that of endothelial cells, thus playing an important role in maintaining glucose metabolism homeostasis in the brain [46]. Early intranasal insulin therapy 24 h after SAH can alleviate glucose metabolism damage, alleviate BBB damage and brain edema, improve neurological dysfunction, and reduce mortality [47]. The decrease in glucose concentration in brain tissue is related to the disruption of brain energy metabolism and poor prognosis after SAH [48]. The anaerobic glycolysis of brain tissue after SAH is also enhanced, and it affects the late-stage neurological function score [49]. Brain microdialysis monitoring revealed an early increase in glutamate levels in brain tissue, followed by a gradual decrease to normal levels. Based on local pyruvate levels and a high lactate-to-pyruvate ratio, mitochondrial dysfunction can be inferred [17]. Other clinical studies have also shown that brain microdialysis monitoring indicators can help indicate mitochondrial dysfunction, which may be the cause of energy metabolism disorders in the brain [50]. After SAH, the expression level of peroxiredoxin-3 (PRDX3) in neuronal mitochondria decreases, and its overexpression can inhibit mitochondrial pathway-mediated neuronal death [51]. Apurinic/antipyrimidic endolucase 1 (APE1) is a protein essential for deoxyribonucleic acid (DNA) repair. A decrease in APE1 levels and an increase in mitochondrial DNA damage and neuronal death were observed in the SAH rat model. APE1 affects mitochondrial apoptosis through the mitochondrial respiratory chain and participates in the process of EBI [52]. 1-azetidin-3-ol maleate (T817MA) is a neurotrophic agent that affects mitochondrial function through the sirtuin 1(SIRT 1) and arc signaling pathways, inhibits lipid peroxidation in EBI after SAH in rats, reduces mitochondrial dysfunction, reduces neuronal apoptosis, and improves neurological prognosis [53]. At present, there is no effective treatment method for mitochondrial dysfunction, and further research is needed.

Existing research has found that the signal pathways involved in brain energy metabolism after SAH include the AMPK, PI3K/Akt, PI3K/Rac/JNK, and glucagon-mediated signaling pathways [54]. For TBI patients, increasing cerebral perfusion pressure (CPP) can improve brain tissue oxygen partial pressure [55], so the increase in cerebral perfusion pressure in EBI after SAH may be beneficial for patients. However, how to timely identify patients who need to increase CPP and determine the optimal CPP will be the focus of future research.

### Destruction of the blood-brain barrier and cerebral edema

Research has shown that matrix metalloproteinase-9 (MMP-9) plays an important role in early blood–brain barrier disruption after SAH [56]. The rat study model showed that basement membrane degradation occurred 6 h after SAH and reached its peak at 48 h, accompanied by upregulation of MMP-9 [54]. In clinical studies, the use of brain microdialysis monitoring has found that local MMP-9 levels in brain tissue increase early after SAH; and are higher in patients with rebleeding, global cerebral edema, and hypoperfusion; and are associated with disease severity and early brain tissue hypoxia [17]. In clinical studies, the increase of MMP-9 in the serum of SAH patients is related to the severity of the disease and the occurrence of cerebral vasospasm [57]. After the blood-brain barrier is disrupted, inflammatory factors can promote brain injury through BBB, leading to cerebral edema. Cerebral edema is an important pathological process after SAH and a significant factor affecting prognosis. Cerebral edema is also a predictive factor for cognitive impairment [58]. Research has shown that cerebral edema after SAH is associated with aquaporin 4 (AQP4). Inhibiting the expression of AQP4 can alleviate cerebral edema, but knocking out the AQP4 gene exacerbates cerebral edema [59]. The excessive activation of nuclear factor E2-related factor 2 (Nrf2) can alleviate the damage to the blood-brain barrier after SAH, promote neuronal survival, and thus improve prognosis [60]. Increased intracranial pressure can lead to damage to the hypothalamus and brainstem, mediating an increase in catecholamine release [61]. Increased intracranial pressure after bleeding can also lead to an increase in carbon dioxide and glutamate, causing activation of astrocytes and the sympathetic nervous system [62]. Overexcitation of the sympathetic nervous system after SAH may exacerbate cerebral edema, reduce cerebral perfusion, and exacerbate EBI [63].

The widely used Fisher scale and modified Fisher scale (mFS) mainly predict large vessel spasms and DCI by quantifying the amount of subarachnoid hemorrhage [64], but their evaluation of cerebral edema is poor. Subarachnoid Hemorrhage Early Brain Edema Score (SEBES) is a new radiological assessment method for EBI brain edema. It is based on head CT and can grade focal edema and global edema, making it an independent predictor of DCI and short-term and long-term adverse prognosis [65]. Based on the assessment of two specific brain tissue levels in each hemisphere (level 1 shows thalamus and basal ganglia at the insular cortex level, level 2 shows central semiovale level), visible sulcus loss or interruption of gray–white matter connectivity is observed. The score ranges from 0 (no edema) to 4 (whole brain edema), which can effectively evaluate the changes from focal edema to whole brain edema. Compared to Hunt–Hess(HH), World Federation of Neurosurgical Societies(WFNS), and mFS grading systems, SEBES may represent changes in microcirculation and therefore has better predictive ability [66]. Early use of MRI examination can also detect cerebral edema and ischemic changes [18]. Therefore, based on neuroimaging techniques, axonal injury and cerebral ischemic changes can be detected after SAH, which can quantify EBI and help identify high-risk patients. More basic molecular research is still needed in the future to clarify the precise mechanisms of brain edema, and clinical studies are also needed to determine the role of these mechanisms in clinical manifestations.

### Neuroinflammation

Current research suggests that neuroinflammation is a normal response of brain tissue to primary injury. After SAH, lymphocytes and macrophages will invade blood vessels and brain tissue. Neutrophils also infiltrate cerebral blood vessels and brain parenchyma, and inhibiting neutrophil activation can reduce early microvascular and brain parenchymal damage in SAH [67]. In the EBI experiment after SAH, the pro-inflammatory cytokine tumor necrosis factor-α (TNF-α), interleukin–1β (IL-1β), and interleukin–6 (IL-6) levels will increase, which can lead to BBB damage [68]. The Ras-MAPK-NF-κB, JAK/STAT, and TLR4/NF-κB signaling pathways are involved in this process [69]. Inhibiting NF-κB gene translocation in experimental SAH mice can alleviate vascular inflammatory response and thus alleviate brain edema [70]. The damage-associated molecular pattern (DAMP) after SAH can induce the release of high mobility group box 1 (HMGB1), exacerbating brain injury and further activating the TLR/NF-κB signaling pathway [71]. Mouse experiments have shown that stress-induced glial cell boundaries can be formed on the first day after SAH, and pericytes regulate the formation of astrocyte glial boundaries through the EphA4/EphrinB2 signaling pathway, inhibiting inflammatory cell infiltration and improving neurological function [72]. Protein tyrosine phosphatase 1B inhibitor (PTP1B-in-1) can regulate the IRS-2/AKT signaling pathway, improve neuroinflammation and neuronal apoptosis, and exert neuroprotective functions [73]. In animal experiments, it was found that takinib alleviates EBI by targeting the inhibition of the TAK1-ROS-NLRP3 inflammasome signaling pathway [74]. Inhibiting miR-26b can alleviate EBI by mediating the upregulation of KLF4/downregulation of STAT3/downregulation of HMGB1 signaling pathways [75]. The combination of HMGB1 and receptors for advanced glycation end products (RAGE) leads to inflammatory response. In the early stage of SAH patients, the levels of HMGB1 and RAGE in the cerebrospinal fluid are significantly increased, and their concentrations are positively correlated with the severity of the disease. Therefore, they may become potential biomarkers for poor prognosis [76]. The use of brain microdialysis monitoring technology in clinical studies has found that the neuroinflammatory marker IL-6 is associated with DCI and poor prognosis after SAH [77]. MMP-9 also participates in neuroinflammation, promotes EBI, and is associated with apoptosis of hippocampal neurons in rats [78].

The main active ingredient of traditional Chinese medicine Asteraceae is eupatilin, which can significantly reduce IL-1β, IL-6, and TNFα, and the inhibit TLR4/MyD88/NF-κB signaling pathway reduces blood-brain barrier damage and brain edema and improves EBI [79]. Alpha asarone (ASA), extracted from the Chinese medical herb Acorus tatarinowii Schott, can promote NR2B/CaMKII interactions, further activate the CREB/BDNF/TrkB signaling pathway, and improve neural function in rats [80]. Animal experiments in SAH have shown that anti-inflammatory drug therapy can reduce blood-brain barrier damage and brain edema and reduce neuronal apoptosis [81]. Clinical studies have also shown that anti-inflammatory therapy may improve patient prognosis [82].

Microglia and immune cells are involved in the neuroinflammatory process and the release of various inflammatory factors, thus playing an important role in the pathophysiological process of EBI. Studies have observed activation of microglia and infiltration of immune cells after SAH [83]. Activation of microglia and infiltration of immune cells can also promote the release of inflammatory factors and reactive oxygen species, leading to tissue damage and neuronal death [84]. Sinomenine (SIN) can upregulate the expression of heme oxygenase-1 (HO-1) and quinine oxidoreductase-1 (NQO-1) by activating the Nrf2 pathway, thereby inhibiting the inflammatory response of microglia after SAH, reducing brain edema and neuronal apoptosis [85]. Glycoprotein non-metastatic melanoma protein B (GPNMB) is widely expressed in neurons, microglia, and astrocytes, which significantly increases after SAH by activating AMPK/NF-κB signaling pathway, inhibiting p-NF-κB, IL-1β, IL-6, and TNFα, then reduce blood-brain barrier damage and neuroinflammation, alleviate brain edema, and improve neurological function in SAH mice [86]. Animal experiments have shown that traditional Chinese medicine electroacupuncture therapy can exert anti-inflammatory effects and reduce neuronal apoptosis by regulating the polarization of microglia, alleviating early brain injury after SAH [87]. Low-dose lipopolysaccharide (LPS) activates the IL-10/IL-10R1 signaling pathway through the deubiquitination of FOXO1 mediated by USP19, regulates microglial polarization, reduces brain edema, blood-brain barrier disruption, neuroinflammation, etc., and has a protective effect on EBI [88].

Macrophage migration inhibitory factor (MIF) is a pro-inflammatory factor that can activate the inflammatory response of the central nervous system. It can activate astrocytes to release inflammatory factors, promote neuronal death, and lead to brain injury [89]. Clinical studies have also found a significant increase in serum MIF concentration in SAH patients, and it is an independent predictor of poor 6-month prognosis in patients [89]. Another study also demonstrated a significant increase in serum MIF in patients with DCI, which has a stronger predictive ability for DCI compared to C-reactive protein and IL-6 [90]. Other clinical studies have found that during the EBI stage after SAH, the levels of IL-6, IL-10, and macrophage inflammatory proteins (MIPβ) in the peripheral blood increase, which are correlated with the severity of the disease and can serve as important biomarkers for EBI [91]. Therefore, the discovery of blood or brain-derived biomarkers can further enhance the understanding of the pathological and physiological processes of EBI, promote the diagnosis and treatment of patients, and improve prognosis.

### Oxidative stress

The degradation products of hemoglobin after SAH can cause oxidative stress, further promoting lipid peroxidation and triggering an inflammatory cascade reaction [92]. Research has shown that reducing oxidative stress can alleviate early brain damage [93]. Rat experiments have shown that traditional Chinese medicine Wu zhu yu reaction (WZYD) activates the Nrf2/heme oxygenase-1 (HO-1) signaling pathway through sirtuin 6 (SIRT6) mediated deacetylation of histone H3 lysine 56 (H3K56), inhibits oxidative stress, and can alleviate cerebral hemorrhage and edema after SAH, thereby alleviating EBI after SAH [94]. Another animal experiment showed that the mitochondrial-targeted antioxidant peptide elamipretide (also known as SS31) reduces lipid peroxidation, increases antioxidant enzyme activity, reverses mitochondrial dysfunction; and reduces blood-brain barrier damage, brain edema, and cell apoptosis after SAH, thereby improving neurological function damage by activating the Nrf2 signaling pathway [95]. The mitochondrial endothelin ligand NL-1 induces mitochondrial autophagy related to the PINK1/PARKIN signaling pathway through mitoNEET, thereby alleviating oxidative stress and cell apoptosis in EBI [96].

### Cortical spreading depolarizations

After SAH, there are significant changes in the concentration of sodium and potassium ions in brain cells, which may cause disturbances in neural electrical activity and cortical spreading depolarizations. CSD is also affected by elevated levels of oxygenated hemoglobin and extracellular potassium ions and decreased levels of NO, glutamate, and endothelin-1 [97]. It may be one of the causes of early brain injury after SAH. Under conditions of ischemia and hypoxia, CSD can lead to cerebral vasoconstriction and decreased cerebral blood flow [98]. The possible mechanisms of CSD include microcirculatory dysfunction after SAH and increased extracellular potassium ion and excitatory amino acid concentrations caused by neuronal energy metabolism disorders [99]. A decrease in NO concentration can also lead to cortical spreading depolarizations, promoting the occurrence of DCI [100].

Early brain injury may be an important cause of secondary brain injury after SAH, but the specific pathological and physiological mechanisms are rarely studied in humans. More research is needed in the future to discover the different clinical manifestations and specific mechanisms of primary and secondary brain injury, in order to develop targeted treatment measures for secondary brain injury.

**Fig. 1:**
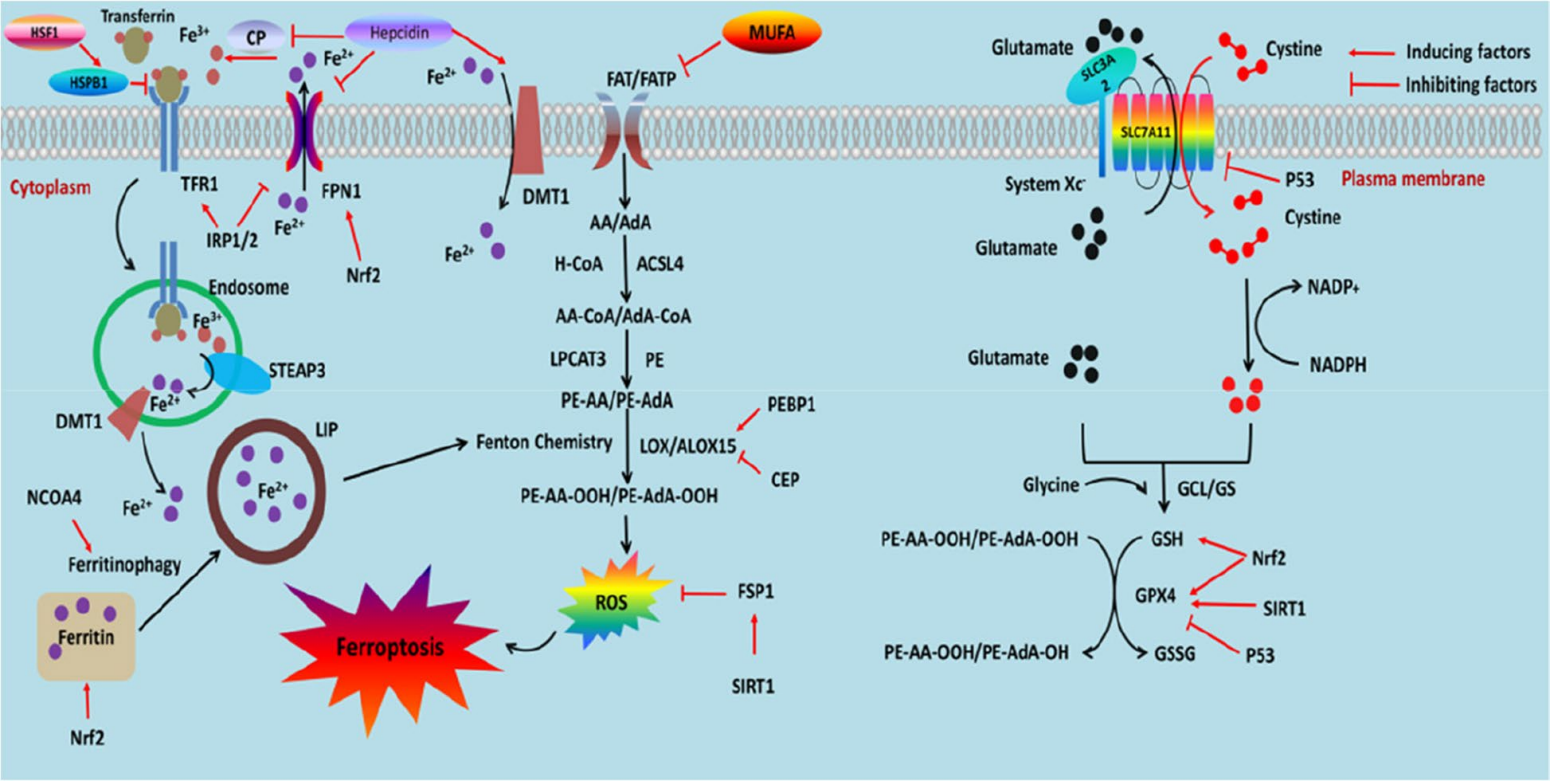
The main pathways related to ferroptosis in EBI after SAH. Extracellular Fe^3+^ mainly binds to TRF and enters cells through TFR1, forming endosomes. HSF1 can inhibit the activity of TFR1 and reduce Fe^3+^ influx by activating HSPB1. Fe^3+^ in the endosome is reduced to Fe^2+^ by STEAP3, and Fe^2+^ is transported to cells through DMT1. Intracellular free iron can be stored in ferritin or transported out of cells through FPN1. NCOA4 mediates ferrithinophage to increase intracellular free iron. Free Fe^2+^ mediates the Fenton reaction to produce reactive oxygen species. FPN1 can reduce intracellular iron concentration by transporting Fe^2+^ out of cells. Hepcidin can inhibit CP’s oxidation of Fe^2+^ to Fe^3+^, promote DMT1’s transport of Fe^2+^ into cells, and inhibit FPN1’s transport of Fe^2+^ out of cells, resulting in an increase in intracellular iron concentration. GSH/GPX4 and FSP1, as the main antioxidant systems, inhibit the production of reactive oxygen species by lipid peroxides. SIRT1 can promote the expression of FSP1 and GPX4, enhancing antioxidant capacity. AA/AdA is absorbed into cells through FAT/FATP, and under the catalysis of ACSL4, CoA, and AA (AdA) are linked to form coenzyme AA-CoA (AdA-CoA) intermediates, which are then catalyzed by LPCAT3 to form arachidonic acid phosphatidylethanolamide (PE-AA) (PE-AdA). PE-AA (PE-AdA) catalyzes the formation of PE-AA-OOH (PE-AdA-OOH) through LOX/ALOX15, producing reactive oxygen species to promote ferroptosis. MUFA can competitively inhibit the function of FAT/FATP. PEBP1 promotes the activity of LOX/ALOX15, increases reactive oxygen species, and promotes ferroptosis, while CEP inhibits LOX/ALOX15 activity. System Xc^**−**^ transfers cysteine into the cell and transfers an equal amount of glutamate out of the cell. Cysteine enters cells and is reduced to cysteine by consuming NADPH. Glutathione (GSH) is synthesized from glutamate, cysteine, and glycine under the catalysis of glutamate cysteine ligase (GCL) and glutathione synthase (GS). Glutathione, as an electron donor, reduces toxic phospholipid hydroperoxides to non-toxic phospholipid alcohols, thereby inhibiting lipid peroxidation. P53 exerts its effect by inhibiting the functions of SLC7A11 and GPX4. Nrf2 can promote ferritin binding to Fe^2+^, promote FPN1 to transport Fe^2+^ out of cells, and thus reduce intracellular iron. It can also activate downstream GSH and GPX4 to inhibit lipid peroxidation. ROS reactive oxygen species, TFR1 transferrin receptor 1, DMT1 divalent metal transporter 1, FPN ferroportin, CP ceruloplasmin, IRP1/2 iron regulatory proteins, ACSL4 acyl CoA synthetase long chain family member 4, LPCAT3 lysophosphatidylcholine acyltransferase 3, AA arachidonic acid, AdA adrenic acid, FAT fatty acid translocase, FATP fatty acid transport protein, PE phosphatidylethanolamine, PE-AA arachidonic acid-phosphatidylethanolamines, PE-AdA adrenic acid-phosphatidylethanolamines, MUFA monounsaturated fatty acid, PEBP1 phosphatidylethanolamine-binding protein 1, ALOX15 arachidonic acid-15-lipoxygenase, CEP cepharanthine, GSH glutathione, GCL glutamatecysteine ligase, GS glutathione synthetase, SLC3A2 solute carrier family 3 member 2, SLC7A11 solute carrier family 7 member 11, GPX4 glutathione peroxidase 4, FSP1 ferroptosis suppressor protein 1, NADPH nicotinamide adenine dinucleotide phosphate hydride, Nrf2 NF-E2-related factor 2, HSF1 heat shock factor 1, HSPB1 heat shock protein B1, NCOA4 nuclear receptor coactivator 4, SIRT1 sirtuin 1

Ferroptosis is a new programmed cell death mechanism, which is characterized by iron-dependent lipid peroxide accumulation-induced cell death [101], and plays an important role in a variety of diseases. The unique morphological features of ferroptosis are mitochondrial shrinkage, loss of mitochondrial cristae, and increased membrane density [102]. Its main biochemical feature is an increase in intracellular free iron and lipid reactive oxygen species (ROS), cystine-glutamate antiporter pathway, reduction of cysteine uptake, and synthesis of glutathione (GSH) [103]. Recent studies have shown that it is closely related to neurological diseases. Ferroptosis occurs in neurons around the hematoma after intracerebral hemorrhage [104]. Ferroptosis is present in early brain injury after subarachnoid hemorrhage and reduced lipid peroxides alleviate ferroptosis (Fig. 1) [30].

## Iron metabolism

Disturbance of iron metabolism plays an important role in SAH [105], and an abnormal increase in intracellular ferrous iron is the initiating factor of ferroptosis [106]. The regulatory system of cellular iron metabolism is complex, involving a variety of proteins to jointly regulate the input, storage, and output of iron, mainly including transferrin receptor 1 (TFR1), divalent metal transporter 1(DMT1), and ferroportin (FPN) [107]. Extracellular Fe^2+^ is mainly oxidized by ceruloplasmin to Fe^3+^, thus maintaining an inactive state. Iron is mainly stored and transferred in the form of inactive ferritin complexes. The extracellular matrix is mainly composed of Fe^3+^, which binds to transferrin (TRF), and then TFR1 mediates iron uptake in most neurons by binding to transferrin on the cell surface, and transferrin-bound iron will then be internalized through clathrin-mediated endocytosis [108]. The six transmembrane epithelial antigen of prostate 3 (STEAP3) reduces Fe^3+^ to Fe^2+^ in the endosome and is then transported by DMT1 to the labile iron pool (LIP). Excessive intracellular iron can be stored in ferritin or transported out of cells through FPN. Usually, intracellular iron is stored in ferritin and closely bound to ferritin, while enhanced degradation of ferritin can promote ferroptosis [109]. FPN is the only known protein that exports intracellular iron in mammals [110]. It is embedded in the whole nerve cell and exports iron through the basolateral membrane and iron homeostasis is severely disturbed in FPN-deficient mice [111]. DMT1 protein promotes iron uptake from cell membranes and transports iron across endosomal membranes in almost all cell types that take up iron via the transferrin/transferrin receptor 1 pathway [112]. DMT1 also transports iron from endosomes to the cytoplasm, releasing intracellular iron and eventually inducing ferroptosis [113]. Disordered iron metabolism can lead to excessive activity of Fe^2+^ in cells, which generates free radicals through the Fenton reaction and participates in oxidative stress and lipid peroxidation, thereby damaging proteins, lipids, and DNA, affecting normal cell function, and ultimately leading to neuronal death [114]. In a rodent SAH study, deferoxamine treatment reduced mortality and third-day neuronal death [28].

The iron-regulating hormone hepcidin can promote iron accumulation by inhibiting FPN1 and ceruloplasmin and activating DMT1 in the cerebral cortex and hippocampus [115]. The expression of hepcidin and DMT1 is increased in the early brain injury of SAH [116], while the expression of FPN1 is decreased [113]. Iron regulatory proteins 1 and 2 (IRP1/2) are the main regulators of cellular iron homeostasis [117], and they play an important role in intracellular iron homeostasis under iron deficiency through the following pathways: (1) stabilize TFR1, (2) inhibit FPN1, (3) mobilize cellular iron reserves [118]. Multiple studies have reported that TFR1 and DMT1 are upregulated, while FPN and IRP1/2 are downregulated after ferroptosis [108]. The decrease in IRP2 expression will limit iron transmission in neurons. IRP2 knockout mice showed increased cell viability in tissues around hematoma after intracerebral hemorrhage, suggesting that IRP2 may become a new target for ferroptosis therapy [119]. However, more research is needed in the future to confirm the specific mechanism and downstream effectors of iron accumulation-induced ROS production.

## Lipid metabolism

As an important part of cellular lipid metabolism, fatty acids have many functions, such as energy supply, cell membrane formation, and signal molecule precursors. Polyunsaturated fatty acids (PUFAs) participate in the synthesis of fat signaling pathways, membrane phospholipid composition, and ferroptosis signal transduction after lipid oxidation, thereby inducing ferroptosis in cells [120]. There are two main pathways for lipid peroxidation after SAH, non-enzymatic pathway and enzymatic pathway. Non-enzymatic lipid peroxidation is mediated by the Fenton reaction. The Fenton reaction occurs between hydrogen peroxide and Fe2+, which can generate hydroxyl radicals (OH-) and participate in the oxidation of PUFA [121]. The enzymatic pathway of lipid peroxidation is regulated by the activity of lipoxygenase, with arachidonic acid-15-lipoxygenase(ALOX15) playing a major role [122]. Acyl CoA synthetase long chain family member 4(ACSL4) is a key enzyme in fatty acid metabolism and is essential for oxidizing cell membrane phospholipids [123]. PUFAs are integrated into the cell membrane by acyl CoA synthetase long chain family member 4 (ACSL4) and lysophosphatidylcholine acyltransferase 3 (LPCAT3), so the PUFAs in the cell membrane are the main targets of reactive oxygen species and the main substrates of lipid peroxidation during ferroptosis [124]. Lipid radicals (L-) combine with O_2_ to form lipid peroxyl radicals (LOO-), which then abstract hydrogen from adjacent PUFAs to form lipid hydrogen peroxide (LOOH) and new lipid radicals, and another occurs oxidation reaction [125]. Malondialdehyde (MDA) produced by lipid peroxidation has mutagenic effects, while 4-hydroxy-2-nonenal (4-HNE) is toxic [126].

The most common PUFA in the body is arachidonicacid (AA), which exists in all tissues [127]. AA and adrenicacid (AdA) are taken up by fatty acid translocase (FAT) and fatty acid transport protein (FATP), which are the main polyunsaturated fatty acids that induce cell ferroptosis [128]. Monooxidized PUFA-OOH cannot mediate ferroptosis in vivo and in vitro, and only when PUFAs are esterified to form membrane phospholipids (PUFA-PLs) can they be oxidized and mediate ferroptosis [128]. ACSL4 catalyzes the connection of coenzyme A and AA (AdA) to form a coenzyme AA-CoA (AdA-CoA) intermediate, which is esterified to phosphatidylethanolamine (PE) by LPCAT3 to form arachidonic acid-phosphatidylethanolamines (PE-AA) (PE-AdA). PE-AA (PE-AdA) can form PE-AA-OOH (PE-AdA-OOH) through lipoxygenase (LOX) in an enzymatic manner or through autoxidation, eventually leading to cell death [129], this reaction occurs on the mitochondrial membrane [101] or on the membrane of mitochondria and endoplasmic reticulum [128]. Exogenous monounsaturated fatty acid (MUFA), such as oleic acid, can effectively inhibit erastin-induced ferroptosis by competing with PUFAs for entry into phospholipids [130].

Phosphatidylethanolamine-binding protein 1 (PEBP1) immobilizes arachidonic acid-15-lipoxygenase (ALOX15) on the cell membrane, thereby increasing the catalytic effect of ALOX15, PE-AA-15-OOH is catalyzed by ALOX15 as a ferroptosis signal in a traumatic brain injury model [122]. In the SAH model, ferroptosis occurred in endothelial cells and microglia, and the level of ALOX15 protein began to rise 24 h after SAH and remained at a high level throughout the EBI period, and the up regulation of ALOX15 may promote ferroptosis [131]. Cepharanthine can protect against brain injury after SAH by inhibiting ALOX15-mediated ferroptosis of endothelial cells and microglia [131]. Silencing the lipoxygenase gene can make cells resist ferroptosis induced by erastin [129].

ACSL4 is a key enzyme in the process of ferroptosis. It alters the sensitivity of cells to ferroptosis by affecting the lipid composition of the cell membrane. The reduction of ACSL4 can increase the resistance of cells to ferroptosis [132]. After SAH, the expression of ACSL4 in rat brains increased significantly, and EBI was partially alleviated by reducing the expression of ACSL4 [133].

## Amino acid metabolism and the cystine-glutamate antiporter pathway (System X_C_^−^)

Subarachnoid hemorrhage changes the metabolism of carbohydrates, lipids, and amino acids in cerebrospinal fluid metabonomics [134]. Ferroptosis is closely related to amino acid metabolism [102]. The production of glutamate in the brain depends on the interaction between neurons and glial cells, and glutamate level may be a regulator of ferroptosis [135]. SAH and secondary tissue ischemia can induce ROS and pro-inflammatory cytokines, damage arterial, capillary, and venous endothelial cells; activate inflammatory cells and platelets; and lead to microthrombosis [136]. Platelet mediated microthrombosis can release glutamate [136, 137]. Activated astrocytes, microglia, and neutrophils synthesize and release glutamate massively after SAH, [8] glutamate levels in cerebrospinal fluid of rats increased within 72 h after SAH, leading to the destruction of the blood-brain barrier and neuronal apoptosis [15]. Clinically, brain glutamate levels rise within minutes after aneurysmal subarachnoid hemorrhage, and elevated glutamate levels 1 to 7 days after SAH are independent predictors of DCI and poor clinical prognosis at 12 months [138].

Glutathione (GSH) is synthesized by glutamic acid, cysteine, and glycine under the catalysis of glutamatecysteine ligase (GCL) and glutathione synthetase (GS) and is involved in the regulation of ferroptosis [139]. Glutathione acts as an electron donor to reduce toxic phospholipid hydroperoxides to non-toxic phospholipid alcohols [121], thereby inhibiting lipoxygenase-mediated lipid peroxidation and playing a key role in preventing ferroptosis [140]. System X_C_^−^ is a cystine-glutamate antiporter, which includes the regulatory subunit solute carrier family 3 member 2 (SLC3A2) and the catalytic subunit solute carrier family 7 member 11 (SLC7A11), that promotes the exchange of cystine and glutamate on the cell membrane, and cysteine can be transferred into cells and an equal amount of glutamate can be transferred out of cells. Cystine is reduced to cysteine by consuming NADPH after entering the cell [101, 141]. Compared with normal mice, mice with knockout of the System X_C_^−^ gene have significantly lower levels of glutamate around neurons, less drug-induced neurotoxicity, and the ability to resist neurotoxic damage [142].

## GPX4

Glutathione peroxidase 4 (GPX4) belongs to the glutathione peroxidase (GPXs) family, but compared with other GPXs, GPX4 lacks a dimerization interface and exists in the form of a monomer. It is a key protein that inhibits ferroptosis. GPX4 is a multifunctional protein that can reduce lipid peroxide in free form or in the form of complexes with lipids(e.g., PLS), proteins(e.g., lipoproteins), or intramembrane complexes [143]. The catalytic reaction involving GPX4 is a recyclable antioxidant pathway that sustainably reduces lipid peroxides (LPO). Firstly, LPO oxidizes the selenol (-SeH) at the active center of GPX4 to selenic acid (-SeOH). Then, the first GSH reduces -SeOH to selenide (-SeSG), and the second GSH further reduces to SeH. The two GSHs generate a glutathione disulfide (GS-SG), forming a complete cycle [121]. Therefore, the activity of GPX4 can be inhibited by inhibiting GSH generation, increasing GSH consumption, and inhibiting selenium metabolism. Dysregulation of the cystine-glutamate antitransport system, antisulfation pathway, and mevalonate pathway reduces GPX4 activity and increases intracellular lipid ROS levels leading to ferroptosis [144]. Ferroptosis suppressor protein 1 (FSP1) can directly eliminate lipid ROS to inhibit ferroptosis independent of GPX4 [145]. NAPDH can promote the clearance of lipid peroxide by acting as an intracellular reductant, so NADPH level can predict the sensitivity of cells to ferroptosis [146]. The results showed that the expression of GPX4 decreased and the level of lipid peroxidation increased in the brain of rats 24 h after SAH; overexpression of GPX4 could reduce lipid peroxidation, inhibit neuronal death, and then improve brain edema and neurological damage in rats [147]. In the SAH rat experiment, quercetin (QCT) downregulated TFR1 by increasing the expression of GPX4, SLC7A11 (xCT), and FPN1, thereby inhibiting neuronal ferroptosis and alleviating EBI injury [148]. Therefore, the reduction of GPX4 expression may play an important role in neuronal ferroptosis in early brain injury, while its overexpression is neuroprotective [147].

## FSP1-CoQ10- NADPH pathway

Ferroptosis suppressor protein 1 (FSP1), also known as Apoptosis-inducing factor mitochondria associated 2 (AIFM2), is a p53 mediated pro-apoptotic gene [149]. FSP1 can reduce CoQ10 to CoQ10H2, which uses NADPH to capture LPO and inhibit lipid peroxidation, thereby inhibiting ferroptosis. This is a new FSP1 CoQ10 NADPH pathway that does not require GPX4 or GSH [149]. In the EBI animal model after SAH, it was found that FSP1 and CoQ10 were significantly reduced, while Fer-1 could increase FSP1 and weaken ferroptosis. Therefore, FSP1 played an important role in the occurrence of ferroptosis after EBI [150]. Therefore, FSP1 may become a new therapeutic target for EBI.

## Nrf2

Nuclear factor E2-related factor 2 (Nrf2) is an important regulator of the cellular antioxidant defense system. It binds to a specific DNA site (antioxidant response element, ARE) to regulate the transcription of a series of antioxidant enzymes [151]. Target genes of Nrf2 play important roles in ferroptosis, including ferritin heavy chain 1, FPN1, GSH, and GPX4. The mechanism of Nrf2 inhibiting ferroptosis: (1) Nrf2 can enhance the function of the antioxidant system by promoting the expression of GSH and GPx4; (2) Nrf2 can simultaneously store and export cellular free iron by promoting the expression of ferritin and FPN1, reducing iron accumulation and preventing ferroptosis. Nrf2 expression was upregulated in the basilar artery of rats after SAH and was significantly activated in neurons, astrocytes, leukocytes, microglia, endothelin cells, smooth muscle cells, and adventitia cells [152]; Nfr2 deficiency increases brain edema and neuronal death 24 h after SAH [153]. The mouse SAH experiment showed that Netrin-1 inhibits neuronal ferroptosis by enhancing peroxisome proliferator-activated receptor gamma (PPARγ), Nrf2, and GPX4 expression [154]. In the rat SAH model, endogenous antioxidant melatonin improves neural function by activating the Nrf2 signaling pathway and downstream HO-1/NQO1 genes, inhibiting neuronal ferroptosis [155].

Kelch-like ECH-associated protein 1 (KEAP1) acts as a “sensor” protein that captures specific metabolic information following SAH and translates it into appropriate responses. Covalent modification of KEAP1 leads to reduced ubiquitination and accumulation of Nrf2 [156]. The Nrf2-ARE pathway is upregulated during 12, 24, and 48 h in the SAH rat model [157]. The treatment of the Nrf2 signaling pathway mainly focuses on covalent small molecule agonists of Keap1. Mitoquinone, promoting mitophagy via the KEAP1/Nrf2/PHB2 pathway, inhibits neuronal death after SAH in rats [158]. In mouse models, increased expression of heme oxygenase-1 in microglia can reduce neuronal death, vasospasm, and cognitive impairment [159]. Heat shock protein 22 (HSP32) regulates the mitochondrial biogenesis triggered by TFAM/Nrf1 through positive feedback, thus playing a neuroprotective role and further weakening oxidative stress and early brain injury [160].

## Ferrithinophage

Cell iron is stored in a non-toxic form in ferritin, which forms a protein complex within the cell, consisting of 24 subunits of the heavy and light chains of ferritin, capable of chelating up to 4500 iron atoms [161], thereby protecting the cell from the influence of free iron Fenton reaction [162]. The selective autophagic degradation of ferritin is known as ferrithinophage, in which nuclear receptor coactivator 4 (NCOA4) acts as a selective autophagic receptor, binds to ferritin and transports it to the lysosome, where ferritin is degraded and iron is released for use by cells [109]. NCOA4-ferritin complexes can be transported to lysosomes through classic ATG8-dependent autophagy pathways and nonclassical ESCRT-mediated pathways such as TAX1BP1, VPS34, ATG9A, and ULK1/2-FIP200 [109, 163]. NCOA4-mediated ferrithinophage can regulate susceptibility to iron death by promoting the accumulation of iron and reactive oxygen species (ROS) [164, 165]. Studies have shown that NCOA4 plays a key role in maintaining cell iron metabolism, and excessive ferrithinophage can lead to iron overload and ferroptosis [166]. NCOA4 interacts with ferritin to promote its transport to autophages through interaction with ATG8-like proteins such as GABARAP and GABARAPL1, thereby releasing iron; inactivation of NOCA can lead to increased deposition of ferritin in cells [109, 167]. When intracellular iron content is high, NCOA4 degrades depending on the interaction between iron and HERC2 ubiquitin E3 ligase, thereby reducing ferrithinophage; in contrast, when the cell iron content is low, the interaction between NCOA4 and HERC2 decreases, resulting in an increase in ferrithinophage [168]. In the early stage after subarachnoid hemorrhage, autophagic activity in the ipsilateral frontal basal cortex increased significantly, which affected EBI [169], and the activation of the autophagy pathway could play a protective role by reducing early brain injury through anti-apoptotic mechanism [169, 170]. The SAH experiments showed that SAH induced ferroptosis in neurons by activating ferrithinophage and reduced ferroptosis by inhibiting autophagy [171]. However, the specific role and mechanism of NCOA4 in EBI after SAH still need further research and clarification.

## Others

In the process of ferroptosis, the activation of the MAPK pathway and the heat shock factor 1 (HSF-1)-heat shock protein B1 (HSPB1) pathway have a significant role. Inhibition of c-Jun NH2-terminal kinase (JNK) and p38 pathways in the MAPKs pathway can significantly alleviate ferroptosis induced by erastin [172]. The HSF1-HSPB1 pathway inhibits ferroptosis by reducing cellular iron uptake and lipid ROS production through protein kinase C (PKC)-mediated phosphorylation of HSPB1 [173]. The protein CDGSH iron sulfur domain 1 (CISD1, also known as mitochondrial membrane protein) that affects iron metabolism also affects the sensitivity of cells to ferroptosis [174]. Tumor suppressor protein P53 regulates ferroptosis, and activation of p53 may lead to ferroptosis after SAH and aggravate early brain injury by mediating the reduction of SLC7A11 and GPX4 [175]. After SAH, SIRT1 is upregulated in cortical neurons [176]. Activation of sirtuin 1 (SIRT1) reduces lipid peroxidation levels by upregulating the expression of GPX4 and FSP1, which can significantly inhibit ferroptosis, thereby alleviating EBI [150]. The isomer of vitamin K2, menaquinone-4 (MK-4) in brain tissue can upregulate the dihydrorotate dehydrogenase (DHODH) protein by activating SIRT1, reduce GSH, prostaglandin-endoperoxide synthase 2(PTGS2), recombinant nicotinamide adenine dinucleotide phosphate oxidase 1 (NOX1), ROS, restore neuronal mitochondrial membrane potential, and alleviate ferroptosis after SAH [177].

## Ferroptosis of glial cells

Astrocytes are the most abundant central glial cells in the brain. They have a strong iron storage capacity, which can avoid iron overload in neurons, thereby inhibiting iron death in neurons [178]. Brain derived neurotrophic factors in astrocytes can also activate Nrf2, thereby inhibiting neuronal ferroptosis [179]. Overexpression of aquaporin 4 in astrocytes reduces neuronal ferroptosis after SAH [180]. Active astrocytes and activated microglia can promote the iron death of oligodendrocytes [181].

In the brain, oligodendrocytes are the main cells that form the myelin sheath [182], and iron is a necessary cofactor for myelin synthesis. Therefore, the iron content of oligodendrocytes is more than 20 times that of astrocytes [102]. Iron overload can induce ferroptosis in oligodendrocytes [183]. Studies have shown that oligodendrocyte ferroptosis can stimulate microglial activation and neuronal damage [181]. Inhibiting ferroptosis of oligodendrocytes can alleviate white matter damage after spinal cord injury [184], and the white matter damage after SAH is closely related to the severity of EBI [185]. In a mouse hemorrhagic stroke model, ferroptosis is the main form of death of oligodendrocytes, and inhibiting ferroptosis can alleviate white matter damage and promote the recovery of neural function [186]. The Rasd1 gene can activate NCOA4-mediated ferrithinophage to induce ferroptosis in oligodendrocytes in white matter injury after SAH by increasing reactive oxygen species, inflammatory factors, free iron, and NCOA4, as well as reducing GPX4, ferritin, and GSH levels [187].

Microglia are the immune system of the brain [188]. M1 microglia release inflammatory cytokines, causing diffuse inflammation in the brain, and play an important role in secondary brain injury caused by SAH, which can lead to neuronal death; M2 microglia limit inflammation and phagocytosis of tissue fragments, participating in neuroprotection and repair after injury [189]. Some studies have shown that ferroptosis can promote neuroinflammation [190], so early after SAH can promote the activation of M2 microglia, thereby protecting neural function [191]. Inducible nitric oxide synthase promotes the survival of M1 microglia by inhibiting ferroptosis, while inhibiting iNOS expression can promote ferroptosis of M1 microglia, thereby alleviating early brain injury after SAH [192]. Hemin can induce ferroptosis in M2 microglia by upregulating ALOX15 and downregulating GPX4 [131]. In animal experiments with SAH, increased activation of microglia and expression of pro-inflammatory factors in the brain are associated with long-term sensorimotor injury [193].

## Conclusion

The pathophysiological mechanism of early brain injury after subarachnoid hemorrhage is still unclear, so there are no targeted prevention or treatment intervention measures at present. Ferroptosis is involved in the neuronal death and glial cell death of early brain injury and plays an important role. In-depth research is needed to clarify its pathophysiological process, so as to find new therapeutic targets and further improve the prognosis of patients (Table 1).

**Table 1:** Factors that cause ferroptosis in EBI after SAH

Author	Year	Mechanism for the factors	Setting
Gao, S. Q. et al. [147]	2020	Decrease of GPX4 expression	Rats and in vitro model
Cao, Y. et al. [194]	2021	Expression of Gpx4 and ACSL4 and Cox2	Rats and in vitro model
Heinsberg, L. W. et al. [195]	2021	Iron homeostasis pathway DNA Methylation.STEAP3 metalloreductase	Clinical
Kuang, H. et al. [175]	2021	Inhibiting p53-induced ferroptosis	Rats
Li, Y. et al. [30]	2021	Fpn expression and the iron content	Rats and in vitro model
Qu, X. et al. [133]	2021	ACSL4	Rats
Zhang, H. et al. [113]	2021	DMT1 signaling activation. Hepcidin	Rats
Zheng, B. et al. [196]	2021	Autophagy dependent ferroptosis	Rats
Gao, S et al. [131]	2022	15-lipoxygenase-1-mediated microglia and endothelial cell ferroptosis	Rats and in vitro model
Liang, Y. D. et al. [171]	2022	Activation of ferritinophagy	Rats
Liu, Y. et al. [180]	2022	Aquaporin 4 depolarization-enhanced transferrin infiltration	Rats
Qu, W. et al. [192]	2022	Ferroptosis of M1 microglia	Rats
Tao, Q. et al. [197]	2022	Autophagy-dependent ferroptosis in microglia	Rats
Yuan, B. et al. [150]	2022	Activation of SIRT1 alleviates ferroptosis in the early brain injury after subarachnoid hemorrhage	Rats and in vitro model
Chen, J. et al. [154]	2023	Netrin-1 alleviates early brain injury by regulating ferroptosis via the PPARγ/Nrf2/GPX4 signaling pathway following subarachnoid hemorrhage	Rats
Jiao, D. et al. [148]	2023	QCT increased the expression levels of GPX4, xCT, and FPN1, while downregulated TfR1	Rats
Cao, C et al. [198]	2023	Restoring system xc- activity by xCT overexpression inhibited neuronal ferroptosis and improved neurological deficits after experimental subarachnoid hemorrhage	Rats
Ma, S. et al. [155]	2023	Melatonin alleviates early brain injury by inhibiting the NRF2-mediated ferroptosis pathway after subarachnoid hemorrhage	Rats
Zhang, J. et al. [177]	2023	Menaquinone-4 attenuates ferroptosis by upregulating DHODH through activation of SIRT1 after subarachnoid hemorrhage	Rats

## Data Availability

Not applicable.

## Abbreviations

SAH: Subarachnoid hemorrhage
EBI: Early brain injury
BBB: Blood-brain barrier
DCI: Delayed cerebral ischemia
ADC: Apparent diffusion coefficient
MRI: Magnetic resonance imaging
CSD: Cortical spreading depolarizations
eNOS: Endothelial NO synthase
NETs: Neutrophil extracellular traps
u-PA: Urokinase type plasminogen activator
PET: Positron emission tomography
CMRO_2_: Cerebral metabolic rate of oxygen
PRDX3: Peroxiredoxin-3
APE1: Apurinic/antipyrimidic endolucase 1
DNA: Deoxyribonucleic acid
SIRT 1: Sirtuin 1
CPP: Cerebral perfusion pressure
MMP-9: Matrix metalloproteinase-9
AQP4: Aquaporin 4
Nrf2: Nuclear factor E2-related factor 2
mFS: Modified Fisher scale
SEBES: Subarachnoid hemorrhage early brain edema score
TNF-α: Tumor necrosis factor-α
IL-1β: Interleukin–1β
IL-6: Interleukin–6
HMGB1: High mobility group box 1
RAGE: Receptors for advanced glycation end products
ROS: Reactive oxygen species
HO-1: Heme oxygenase-1
NQO-1: Quinine oxidoreductase-1
GPNMB: Glycoprotein non-metastatic melanoma protein B
MIF: Macrophage migration inhibitory factor
MIPβ: Macrophage inflammatory proteins
GSH: Glutathione
TFR1: Transferrin receptor 1
DMT1: Divalent metal transporter 1
FPN: Ferroportin
TRF: Transferrin
STEAP3: Six transmembrane epithelial antigen of prostate 3
LIP: Labile iron pool
IRP1/2: Iron regulatory proteins 1 and 2
PUFAs: Polyunsaturated fatty acids
ALOX15: Arachidonic acid-15-lipoxygenase
ACSL4: Acyl CoA synthetase long chain family member 4
LPCAT3: Lysophosphatidylcholine acyltransferase 3
AA: Arachidonicacid
AdA: Adrenicacid
FAT: Fatty acid translocase
FATP: Fatty acid transport protein
PEBP1: Phosphatidylethanolamine-binding protein 1
GCL: Glutamatecysteine ligase
GS: Glutathione synthetase
GPX4: Glutathione peroxidase 4
FSP1: Ferroptosis suppressor protein 1
KEAP1: Kelch-like ECH-associated protein 1
NCOA4: Nuclear receptor coactivator 4

## References

[1] Lawton MT, Vates GE (2017). Subarachnoid hemorrhage. N Engl J Med.

[2] Long B, Koyfman A, Runyon MS (2017). Subarachnoid hemorrhage: updates in diagnosis and management. Emerg Med Clin North Am.

[3] de Rooij NK, Linn FH, van der Plas JA, Algra A, Rinkel GJ (2007). Incidence of subarachnoid haemorrhage: a systematic review with emphasis on region, age, gender and time trends. J Neurol Neurosurg Psychiatry.

[4] Etminan N, Chang HS, Hackenberg K, de Rooij NK, Vergouwen MDI, Rinkel GJE (2019). Worldwide incidence of aneurysmal subarachnoid hemorrhage according to region, time period, blood pressure, and smoking prevalence in the population: a systematic review and meta-analysis. JAMA Neurol.

[5] Spetzler RF, Zabramski JM, McDougall CG, Albuquerque FC, Hills NK, Wallace RC (2018). Analysis of saccular aneurysms in the Barrow Ruptured Aneurysm Trial. J Neurosurg.

[6] Fang YJ, Mei SH, Lu JN, Chen YK, Chai ZH, Dong X (2019). New risk score of the early period after spontaneous subarachnoid hemorrhage: for the prediction of delayed cerebral ischemia. CNS Neurosci Ther.

[7] Larsen CC, Astrup J (2013). Rebleeding after aneurysmal subarachnoid hemorrhage: a literature review. World Neurosurg.

[8] Geraghty JR, Testai FD (2017). Delayed cerebral ischemia after subarachnoid hemorrhage: beyond vasospasm and towards a multifactorial pathophysiology. Curr Atheroscler Rep.

[9] Peng JH, Qin XH, Pang JW, Wu Y, Dong JH, Huang CR (2017). Apolipoprotein E ε4: a possible risk factor of intracranial pressure and white matter perfusion in good-grade aneurysmal subarachnoid hemorrhage patients at early stage. Front Neurol.

[10] Liu W, Li R, Yin J, Guo S, Chen Y, Fan H (2019). Mesenchymal stem cells alleviate the early brain injury of subarachnoid hemorrhage partly by suppression of Notch1-dependent neuroinflammation: involvement of Botch. J Neuroinflammation.

[11] Sun CM, Enkhjargal B, Reis C, Zhou KR, Xie ZY, Wu LY (2019). Osteopontin attenuates early brain injury through regulating autophagy-apoptosis interaction after subarachnoid hemorrhage in rats. CNS Neurosci Ther.

[12] Kusaka G, Ishikawa M, Nanda A, Granger DN, Zhang JH (2004). Signaling pathways for early brain injury after subarachnoid hemorrhage. J Cereb Blood Flow Metab.

[13] Sabri M, Lass E, Macdonald RL (2013). Early brain injury: a common mechanism in subarachnoid hemorrhage and global cerebral ischemia. Stroke Res Treat.

[14] Cahill J, Calvert JW, Zhang JH (2006). Mechanisms of early brain injury after subarachnoid hemorrhage. J Cereb Blood Flow Metab.

[15] Zhang C, Jiang M, Wang WQ, Zhao SJ, Yin YX, Mi QJ (2020). Selective mGluR1 negative allosteric modulator reduces blood-brain barrier permeability and cerebral edema after experimental subarachnoid hemorrhage. Transl Stroke Res.

[16] Connolly ES, Rabinstein AA, Carhuapoma JR, Derdeyn CP, Dion J, Higashida RT (2012). Guidelines for the management of aneurysmal subarachnoid hemorrhage: a guideline for healthcare professionals from the American Heart Association/American Stroke Association. Stroke.

[17] Helbok R, Schiefecker AJ, Beer R, Dietmann A, Antunes AP, Sohm F (2015). Early brain injury after aneurysmal subarachnoid hemorrhage: a multimodal neuromonitoring study. Crit Care.

[18] Weimer JM, Jones SE, Frontera JA (2017). Acute cytotoxic and vasogenic edema after subarachnoid hemorrhage: a quantitative MRI study. AJNR Am J Neuroradiol.

[19] Claassen J, Carhuapoma JR, Kreiter KT, Du EY, Connolly ES, Mayer SA (2002). Global cerebral edema after subarachnoid hemorrhage: frequency, predictors, and impact on outcome. Stroke.

[20] Coulibaly AP, Provencio JJ (2020). Aneurysmal subarachnoid hemorrhage: an overview of inflammation-induced cellular changes. Neurotherapeutics.

[21] Zhang Z-H, Han Y-L, Wang C-X, Zhou C-H, Wu L-Y, Zhang H-S (2016). The effect of subarachnoid erythrocyte lysate on brain injury: a preliminary study. Biosci Rep.

[22] Chen J, Zheng ZV, Lu G, Chan WY, Zhang Y, Wong GKC (2022). Microglia activation, classification and microglia-mediated neuroinflammatory modulators in subarachnoid hemorrhage. Neural Regen Res.

[23] Schneider UC, Xu R, Vajkoczy P (2018). Inflammatory events following subarachnoid hemorrhage (SAH). Curr Neuropharmacol.

[24] Shuttleworth CW, Andrew RD, Akbari Y, Ayata C, Balu R, Brennan KC (2020). Which spreading depolarizations are deleterious to brain tissue?. Neurocrit Care.

[25] Dreier JP, Lemale CL, Kola V, Friedman A, Schoknecht K (2018). Spreading depolarization is not an epiphenomenon but the principal mechanism of the cytotoxic edema in various gray matter structures of the brain during stroke. Neuropharmacology.

[26] Lauritzen M, Dreier JP, Fabricius M, Hartings JA, Graf R, Strong AJ (2011). Clinical relevance of cortical spreading depression in neurological disorders: migraine, malignant stroke, subarachnoid and intracranial hemorrhage, and traumatic brain injury. J Cereb Blood Flow Metab.

[27] Windmuller O, Lindauer U, Foddis M, Einhaupl KM, Dirnagl U, Heinemann U (2005). Ion changes in spreading ischaemia induce rat middle cerebral artery constriction in the absence of NO. Brain.

[28] Lee JY, Keep RF, He Y, Sagher O, Hua Y, Xi G (2010). Hemoglobin and iron handling in brain after subarachnoid hemorrhage and the effect of deferoxamine on early brain injury. J Cereb Blood Flow Metab.

[29] Kooijman E, Nijboer CH, Van Velthoven CT, Kavelaars A, Kesecioglu J, Heijnen CJ (2014). The rodent endovascular puncture model of subarachnoid hemorrhage mechanisms of brain damage and therapeutic strategies. J Neuroinflammation.

[30] Li Y, Liu Y, Wu P, Tian Y, Liu B, Wang J (2021). Inhibition of ferroptosis alleviates early brain injury after subarachnoid hemorrhage in vitro and in vivo via reduction of lipid peroxidation. Cell Mol Neurobiol.

[31] Rass V, Helbok R (2019). Early brain injury after poor-grade subarachnoid hemorrhage. Curr Neurol Neurosci Rep.

[32] Hviid CVB, Lauridsen SV, Gyldenholm T, Sunde N, Parkner T, Hvas AM (2020). Plasma neurofilament light chain is associated with poor functional outcome and mortality rate after spontaneous subarachnoid hemorrhage. Transl Stroke Res.

[33] Takemoto Y, Hasegawa Y, Hayashi K, Cao C, Hamasaki T, Kawano T (2020). The stabilization of central sympathetic nerve activation by renal denervation prevents cerebral vasospasm after subarachnoid hemorrhage in rats. Transl Stroke Res.

[34] Conzen C, Becker K, Albanna W, Weiss M, Bach A, Lushina N (2019). The acute phase of experimental subarachnoid hemorrhage: intracranial pressure dynamics and their effect on cerebral blood flow and autoregulation. Transl Stroke Res.

[35] Tso MK, Macdonald RL (2014). Subarachnoid hemorrhage: a review of experimental studies on the microcirculation and the neurovascular unit. Transl Stroke Res.

[36] Friedrich B, Müller F, Feiler S, Schöller K, Plesnila N (2012). Experimental subarachnoid hemorrhage causes early and long-lasting microarterial constriction and microthrombosis: an in-vivo microscopy study. J Cereb Blood Flow Metab.

[37] Friedrich B, Michalik R, Oniszczuk A, Abubaker K, Kozniewska E, Plesnila N (2014). CO2 has no therapeutic effect on early microvasospasm after experimental subarachnoid hemorrhage. J Cereb Blood Flow Metab.

[38] Crobeddu E, Pilloni G, Tardivo V, Fontanella MM, Panciani PP, Spena G (2016). Role of nitric oxide and mechanisms involved in cerebral injury after subarachnoid hemorrhage: is nitric oxide a possible answer to cerebral vasospasm?. J Neurosurg Sci.

[39] Lenz IJ, Plesnila N, Terpolilli NA (2021). Role of endothelial nitric oxide synthase for early brain injury after subarachnoid hemorrhage in mice. J Cereb Blood Flow Metab.

[40] Terpolilli NA, Feiler S, Dienel A, Müller F, Heumos N, Friedrich B (2016). Nitric oxide inhalation reduces brain damage, prevents mortality, and improves neurological outcome after subarachnoid hemorrhage by resolving early pial microvasospasms. J Cereb Blood Flow Metab.

[41] Hao X, Zeng Z, Liang L, Feng Z, Li W, Xiong B (2023). The role of neutrophil extracellular traps in early microthrombosis and brain injury after subarachnoid hemorrhage in mice. Transl Stroke Res.

[42] Pisapia JM, Xu X, Kelly J, Yeung J, Carrion G, Tong H (2012). Microthrombosis after experimental subarachnoid hemorrhage: time course and effect of red blood cell-bound thrombin-activated pro-urokinase and clazosentan. Exp Neurol.

[43] Helbok R, Ko S-B, Schmidt JM, Kurtz P, Fernandez L, Choi HA (2011). Global cerebral edema and brain metabolism after subarachnoid hemorrhage. Stroke.

[44] Hayashi T, Suzuki A, Hatazawa J, Hadeishi H, Shirane R, Tominaga T (2008). Post-operative changes of cerebral circulation and metabolism in the acute stage of low-grade aneurysmal subarachnoid hemorrhage. Neurol Res.

[45] Sarrafzadeh AS, Haux D, Lüdemann L, Amthauer H, Plotkin M, Küchler I (2004). Cerebral ischemia in aneurysmal subarachnoid hemorrhage: a correlative microdialysis-PET study. Stroke.

[46] Castro V, Skowronska M, Lombardi J, He J, Seth N, Velichkovska M (2017). Occludin regulates glucose uptake and ATP production in pericytes by influencing AMP-activated protein kinase activity. J Cereb Blood Flow Metab.

[47] Xu LB, Huang HD, Zhao M, Zhu GC, Xu Z (2021). Intranasal insulin treatment attenuates metabolic distress and early brain injury after subarachnoid hemorrhage in mice. Neurocrit Care.

[48] Kurtz P, Claassen J, Helbok R, Schmidt JM, Fernandez L, Presciutti M, Stuart RM, Connolly ES, Lee K, Badjatia N, Mayer SA (2014). Systemic glucose variability predicts cerebral metabolic distress and mortality after subarachnoid hemorrhage: a retrospective observational study. Crit Care.

[49] Barcelos GK, Tholance Y, Grousson S, Renaud B, Perret-Liaudet A, Dailler F (2013). Outcome of poor-grade subarachnoid hemorrhage as determined by biomarkers of glucose cerebral metabolism. Neurocrit Care.

[50] Jacobsen A, Nielsen TH, Nilsson O, Schalén W, Nordström CH (2014). Bedside diagnosis of mitochondrial dysfunction in aneurysmal subarachnoid hemorrhage. Acta Neurol Scand.

[51] Li H, Wang Z, Xie X, Luo M, Shen H, Li X (2023). Peroxiredoxin-3 plays a neuroprotective role in early brain injury after experimental subarachnoid hemorrhage in rats. Brain Res Bull.

[52] Dai K, Wang Z, Gao B, Li L, Gu F, Tao X, et al. APE1 regulates mitochondrial DNA damage repair after experimental subarachnoid haemorrhage in vivo and in vitro. Stroke Vasc Neurol. 2023:002524. 10.1136/svn-2023-002524 PMC11221324 37612054

[53] Chen WW, Sun FQ, Wang B, Tian XX, Zhang RP, Liu WB (2023). T817MA regulates mitochondrial dynamics via Sirt1 and arc following subarachnoid hemorrhage. Neuroscience.

[54] Li X, Zeng L, Lu X, Chen K, Yu M, Wang B (2023). Early brain injury and neuroprotective treatment after aneurysmal subarachnoid hemorrhage: a literature review. Brain Sci.

[55] Johnston AJ, Steiner LA, Coles JP, Chatfield DA, Fryer TD, Smielewski P (2005). Effect of cerebral perfusion pressure augmentation on regional oxygenation and metabolism after head injury*. Crit Care Med.

[56] Guo Z, Xu L, Wang X, Sun X (2015). MMP-9 expression and activity is concurrent with endothelial cell apoptosis in the basilar artery after subarachnoid hemorrhaging in rats. Neurol Sci.

[57] Fischer M, Dietmann A, Beer R, Broessner G, Helbok R, Pfausler B (2013). Differential regulation of matrix-metalloproteinases and their tissue inhibitors in patients with aneurysmal subarachnoid hemorrhage. PLoS One.

[58] Hayman EG, Wessell A, Gerzanich V, Sheth KN, Simard JM (2017). Mechanisms of global cerebral edema formation in aneurysmal subarachnoid hemorrhage. Neurocrit Care.

[59] Higashida T, Kreipke CW, Rafols JA, Peng C, Schafer S, Schafer P (2011). The role of hypoxia-inducible factor-1α, aquaporin-4, and matrix metalloproteinase-9 in blood-brain barrier disruption and brain edema after traumatic brain injury. J Neurosurg.

[60] Sivandzade F, Prasad S, Bhalerao A, Cucullo L (2019). NRF2 and NF-қB interplay in cerebrovascular and neurodegenerative disorders: molecular mechanisms and possible therapeutic approaches. Redox Biol.

[61] Hasegawa Y, Uchikawa H, Kajiwara S, Morioka M (2022). Central sympathetic nerve activation in subarachnoid hemorrhage. J Neurochem.

[62] Kawakita F, Kanamaru H, Asada R, Suzuki Y, Nampei M, Nakajima H (2022). Roles of glutamate in brain injuries after subarachnoid hemorrhage. Histol Histopathol.

[63] Demura M, Ishii H, Takarada-Iemata M, Kamide T, Yoshikawa A, Nakada M (2023). Sympathetic nervous hyperactivity impairs microcirculation leading to early brain injury after subarachnoid hemorrhage. Stroke.

[64] Frontera JA, Claassen J, Schmidt JM, Wartenberg KE, Temes R, Connolly ES, Macdonald RL, Mayer SA (2006). Prediction of symptomatic vasospasm after subarachnoid hemorrhage the modified fisher scale. Neurosurgery.

[65] Ahn SH, Savarraj JP, Pervez M, Jones W, Park J, Jeon SB (2018). The subarachnoid hemorrhage early brain edema score predicts delayed cerebral ischemia and clinical outcomes. Neurosurgery.

[66] Frontera JA, Ahmed W, Zach V, Jovine M, Tanenbaum L, Sehba F (2015). Acute ischaemia after subarachnoid haemorrhage, relationship with early brain injury and impact on outcome: a prospective quantitative MRI study. J Neurol Neurosurg Psychiatry.

[67] Weng W, Cheng F, Zhang J (2022). Specific signature biomarkers highlight the potential mechanisms of circulating neutrophils in aneurysmal subarachnoid hemorrhage. Front Pharmacol.

[68] Sozen T, Tsuchiyama R, Hasegawa Y, Suzuki H, Jadhav V, Nishizawa S (2009). Role of interleukin-1beta in early brain injury after subarachnoid hemorrhage in mice. Stroke.

[69] Galea J, Ogungbenro K, Hulme S, Patel H, Scarth S, Hoadley M (2018). Reduction of inflammation after administration of interleukin-1 receptor antagonist following aneurysmal subarachnoid hemorrhage: results of the subcutaneous interleukin-1Ra in SAH (SCIL-SAH) study. J Neurosurg.

[70] Siler DA, Berlow YA, Kukino A, Davis CM, Nelson JW, Grafe MR (2015). Soluble epoxide hydrolase in hydrocephalus, cerebral edema, and vascular inflammation after subarachnoid hemorrhage. Stroke.

[71] Paudel YN, Angelopoulou E, Piperi C, Othman I, Shaikh MF (2020). HMGB1-mediated neuroinflammatory responses in brain injuries: potential mechanisms and therapeutic opportunities. Int J Mol Sci.

[72] Zhou J, Guo P, Duan M, Li J, Ru X, Li L (2023). EphA4/EphrinB2 signaling mediates pericyte-induced transient glia limitans formation as a secondary protective barrier after subarachnoid hemorrhage in mice. Exp Neurol.

[73] Zhang ZH, Zhou XM, Zhang X (2023). Role of protein tyrosine phosphatase 1b inhibitor in early brain injury of subarachnoid hemorrhage in mice. Brain Sci.

[74] Wang W, Pang C, Zhang J, Peng L, Zhang X, Shi L (2023). Takinib inhibits microglial M1 polarization and oxidative damage after subarachnoid hemorrhage by targeting TAK1-dependent NLRP3 inflammasome signaling pathway. Front Immunol.

[75] Huang Z, Liu J, Xu J, Dai L, Wang H (2023). Downregulation of miR-26b attenuates early brain injury induced by subarachnoid hemorrhage via mediating the KLF4/STAT3/HMGB1 axis. Exp Neurol.

[76] Chu XH, Hu HY, Godje ISG, Zhu LJ, Zhu JB, Feng YL (2023). Elevated HMGB1 and sRAGE levels in cerebrospinal fluid of aneurysmal subarachnoid hemorrhage patients. J Stroke Cerebrovasc Dis.

[77] Mellergård P, Åneman O, Sjögren F, Säberg C, Hillman J (2011). Differences in cerebral extracellular response of interleukin-1β, interleukin-6, and interleukin-10 after subarachnoid hemorrhage or severe head trauma in humans. Neurosurgery.

[78] Guo Z, Sun X, He Z, Jiang Y, Zhang X, Zhang JH (2010). Matrix metalloproteinase-9 potentiates early brain injury after subarachnoid hemorrhage. Neurol Res.

[79] Hong Y, He S, Zou Q, Li C, Wang J, Chen R (2023). Eupatilin alleviates inflammatory response after subarachnoid hemorrhage by inhibition of TLR4/MyD88/NF-κB axis. J Biochem Mol Toxicol.

[80] Gao X, Li R, Luo L, Liao C, Yang H, Mao S. Alpha-asarone ameliorates neurological dysfunction of subarachnoid hemorrhagic rats in both acute and recovery phases via regulating the CaMKII-dependent pathways. Transl Stroke Res. 2023. 10.1007/s12975-023-01139-3. 10.1007/s12975-023-01139-3 36781743

[81] Zhang XS, Zhang X, Wu Q, Li W, Wang CX, Xie GB (2014). Astaxanthin offers neuroprotection and reduces neuroinflammation in experimental subarachnoid hemorrhage. J Surg Res.

[82] Muroi C, Hugelshofer M, Seule M, Keller E (2013). The impact of nonsteroidal anti-inflammatory drugs on inflammatory response after aneurysmal subarachnoid hemorrhage. Neurocrit Care.

[83] Yu Z, Yang L, Yang Y, Chen S, Sun D, Xu H (2018). Epothilone B benefits nigral dopaminergic neurons by attenuating microglia activation in the 6-hydroxydopamine lesion mouse model of parkinson’s disease. Front Cell Neurosci.

[84] Campolo M, Paterniti I, Siracusa R, Filippone A, Esposito E, Cuzzocrea S (2019). TLR4 absence reduces neuroinflammation and inflammasome activation in Parkinson’s diseases in vivo model. Brain Behav Immun.

[85] Fu C, Xin H, Qian Z, Li X, Gao J, Fan Y (2023). Sinomenine protects against early brain injury by inhibiting microglial inflammatory response via Nrf2-dependent pathway after subarachnoid hemorrhage. Brain Sci.

[86] Li T, Zhang Y, Lu Q, Lei L, Du J, Lu X (2023). GPNMB ameliorates neuroinflammation via the modulation of AMPK/NFκB signaling pathway after SAH in mice. J Neuroimmune Pharmacol.

[87] Wang Y, Yang X, Cao Y, Li X, Xu R, Yan J (2023). Electroacupuncture alleviates early brain injury via modulating microglia polarization and suppressing neuroinflammation in a rat model of subarachnoid hemorrhage. Heliyon.

[88] Tao W, Zhang G, Liu C, Jin L, Li X, Yang S (2023). Low-dose LPS alleviates early brain injury after SAH by modulating microglial M1/M2 polarization via USP19/FOXO1/IL-10/IL-10R1 signaling. Redox Biol.

[89] Chen Y-H, Cheng Z-Y, Shao L-H, Shentu H-S, Fu B (2017). Macrophage migration inhibitory factor as a serum prognostic marker in patients with aneurysmal subarachnoid hemorrhage. Clin Chim Acta.

[90] Yang X, Peng J, Pang J, Wan W, Zhong C, Peng T (2019). The association between serum macrophage migration inhibitory factor and delayed cerebral ischemia after aneurysmal subarachnoid hemorrhage. Neurotox Res.

[91] Savarraj J, Parsha K, Hergenroeder G, Ahn S, Chang TR, Kim DH (2018). Early brain injury associated with systemic inflammation after subarachnoid hemorrhage. Neurocrit Care.

[92] Higashi Y, Maruhashi T, Noma K, Kihara Y (2014). Oxidative stress and endothelial dysfunction: clinical evidence and therapeutic implications. Trends Cardiovasc Med.

[93] Endo H, Nito C, Kamada H, Yu F, Chan PH (2006). Reduction in oxidative stress by superoxide dismutase overexpression attenuates acute brain injury after subarachnoid hemorrhage via activation of Akt/glycogen synthase kinase-3β survival signaling. J Cereb Blood Flow Metab.

[94] Xu M, Yue Q, He Z, Ling X, Wang W, Gong M (2024). Wu-zhu-yu decoction reduces early brain injury following subarachnoid hemorrhage in vivo and in vitro by activating the Nrf2 antioxidant system via SIRT6 targeting. J Ethnopharmacol.

[95] Zhou J, Shen R, Makale EC, Zhong W, Chen Z, Huang Q (2023). SS31 confers cerebral protection by reversing mitochondrial dysfunction in early brain injury following subarachnoid hemorrhage, via the Nrf2- and PGC-1α-dependent pathways. Neurochem Res.

[96] Zhang T, Zhang M. NL-1 promotes PINK1-Parkin-mediated mitophagy through MitoNEET inhibition in subarachnoid hemorrhage. Neurochem Res. 2023. 10.1007/s11064-023-04024-5. 10.1007/s11064-023-04024-5 37828361

[97] Dreier JP, Major S, Manning A, Woitzik J, Drenckhahn C, Steinbrink J (2009). Cortical spreading ischaemia is a novel process involved in ischaemic damage in patients with aneurysmal subarachnoid haemorrhage. Brain.

[98] Sukhotinsky I, Dilekoz E, Moskowitz MA, Ayata C (2008). Hypoxia and hypotension transform the blood flow response to cortical spreading depression from hyperemia into hypoperfusion in the rat. J Cereb Blood Flow Metab.

[99] Masuoka T, Ikeda R, Konishi S (2019). Persistent activation of histamine H1 receptors in the hippocampal CA1 region enhances NMDA receptor-mediated synaptic excitation and long-term potentiation in astrocyte- and D-serine-dependent manner. Neuropharmacology.

[100] Petzold GC, Haack S, von Bohlen und Halbach O, Priller J, Lehmann T-N, Heinemann U (2008). Nitric oxide modulates spreading depolarization threshold in the human and rodent cortex. Stroke.

[101] Dixon SJ, Lemberg KM, Lamprecht MR, Skouta R, Zaitsev EM, Gleason CE (2012). Ferroptosis: an iron-dependent form of nonapoptotic cell death. Cell.

[102] Stockwell BR, FriedmannAngeli JP, Bayir H, Bush AI, Conrad M, Dixon SJ (2017). Ferroptosis: a regulated cell death nexus linking metabolism, redox biology, and disease. Cell.

[103] Tang M, Chen Z, Wu D, Chen L (2018). Ferritinophagy/ferroptosis: iron-related newcomers in human diseases. J Cell Physiol.

[104] Zille M, Karuppagounder SS, Chen Y, Gough PJ, Bertin J, Finger J (2017). Neuronal death after hemorrhagic stroke in vitro and in vivo shares features of ferroptosis and necroptosis. Stroke.

[105] Fujii M, Yan J, Rolland WB, Soejima Y, Caner B, Zhang JH (2013). Early brain injury, an evolving frontier in subarachnoid hemorrhage research. Transl Stroke Res.

[106] Wang Z, Ding Y, Wang X, Lu S, Wang C, He C (2018). Pseudolaric acid B triggers ferroptosis in glioma cells via activation of Nox4 and inhibition of xCT. Cancer Lett.

[107] Hentze MW, Muckenthaler MU, Galy B, Camaschella C (2010). Two to tango: regulation of Mammalian iron metabolism. Cell.

[108] Morris G, Berk M, Carvalho AF, Maes M, Walker AJ, Puri BK (2018). Why should neuroscientists worry about iron? The emerging role of ferroptosis in the pathophysiology of neuroprogressive diseases. Behav Brain Res.

[109] Mancias JD, Wang X, Gygi SP, Harper JW, Kimmelman AC (2014). Quantitative proteomics identifies NCOA4 as the cargo receptor mediating ferritinophagy. Nature.

[110] Ganz T (2005). Cellular iron: ferroportin is the only way out. Cell Metab.

[111] Drakesmith H, Nemeth E, Ganz T (2015). Ironing out ferroportin. Cell Metab.

[112] Skjorringe T, Burkhart A, Johnsen KB, Moos T (2015). Divalent metal transporter 1 (DMT1) in the brain: implications for a role in iron transport at the blood-brain barrier, and neuronal and glial pathology. Front Mol Neurosci.

[113] Zhang H, Ostrowski R, Jiang D, Zhao Q, Liang Y, Che X (2021). Hepcidin promoted ferroptosis through iron metabolism which is associated with DMT1 signaling activation in early brain injury following subarachnoid hemorrhage. Oxid Med Cell Longev.

[114] Ward RJ, Zucca FA, Duyn JH, Crichton RR, Zecca L (2014). The role of iron in brain ageing and neurodegenerative disorders. Lancet Neurol.

[115] Li L, Holscher C, Chen BB, Zhang ZF, Liu YZ (2011). Hepcidin treatment modulates the expression of divalent metal transporter-1, ceruloplasmin, and ferroportin-1 in the rat cerebral cortex and hippocampus. Biol Trace Elem Res.

[116] Tan G, Liu L, He Z, Sun J, Xing W, Sun X (2016). Role of hepcidin and its downstream proteins in early brain injury after experimental subarachnoid hemorrhage in rats. Mol Cell Biochem.

[117] Wang W, Di X, D’Agostino RB, Torti SV, Torti FM (2007). Excess capacity of the iron regulatory protein system. J Biol Chem.

[118] Bayeva M, Khechaduri A, Puig S, Chang HC, Patial S, Blackshear PJ (2012). mTOR regulates cellular iron homeostasis through tristetraprolin. Cell Metab.

[119] Chen M, Awe OO, Chen-Roetling J, Regan RF (2010). Iron regulatory protein-2 knockout increases perihematomal ferritin expression and cell viability after intracerebral hemorrhage. Brain Res.

[120] DeBose-Boyd RA (2018). Significance and regulation of lipid metabolism. Semin Cell Dev Biol.

[121] Forcina GC, Dixon SJ (2019). GPX4 at the crossroads of lipid homeostasis and ferroptosis. Proteomics.

[122] Wenzel SE, Tyurina YY, Zhao J, St Croix CM, Dar HH, Mao G (2017). PEBP1 Wardens ferroptosis by enabling lipoxygenase generation of lipid death signals. Cell.

[123] Li D, Li Y (2020). The interaction between ferroptosis and lipid metabolism in cancer. Signal Transduct Target Ther.

[124] Dixon SJ, Winter GE, Musavi LS, Lee ED, Snijder B, Rebsamen M (2015). Human haploid cell genetics reveals roles for lipid metabolism genes in nonapoptotic cell death. ACS Chem Biol.

[125] Rice-Evans C, Burdon R (1993). Free radical-lipid interactions and their pathological consequences. Prog Lipid Res.

[126] Ayala A, Muñoz MF, Argüelles S (2014). Lipid peroxidation: production, metabolism, and signaling mechanisms of malondialdehyde and 4-hydroxy-2-nonenal. Oxid Med Cell Longev.

[127] Janssen CI, Kiliaan AJ (2014). Long-chain polyunsaturated fatty acids (LCPUFA) from genesis to senescence: the influence of LCPUFA on neural development, aging, and neurodegeneration. Prog Lipid Res.

[128] Kagan VE, Mao G, Qu F, Angeli JPF (2016). Oxidized arachidonic and adrenic PEs navigate cells to ferroptosis. Nat Chem Biol.

[129] Yang WS, Kim KJ, Gaschler MM, Patel M, Shchepinov MS, Stockwell BR (2016). Peroxidation of polyunsaturated fatty acids by lipoxygenases drives ferroptosis. Proc Natl Acad Sci U S A.

[130] Magtanong L, Ko PJ, To M, Cao JY, Forcina GC, Tarangelo A (2019). Exogenous monounsaturated fatty acids promote a ferroptosis-resistant cell state. Cell Chem Biol.

[131] Gao S, Zhou L, Lu J, Fang Y, Wu H, Xu W (2022). Cepharanthine attenuates early brain injury after subarachnoid hemorrhage in mice via inhibiting 15-lipoxygenase-1-mediated microglia and endothelial cell ferroptosis. Oxid Med Cell Longev.

[132] Doll S, Proneth B, Tyurina YY, Panzilius E, Kobayashi S, Ingold I, Irmler M, Beckers J, Aichler M, Walch A, Prokisch H (2017). ACSL4 dictates ferroptosis sensitivity by shaping cellular lipid composition. Nat Chem Biol.

[133] Qu XF, Liang TY, Wu DG, Lai NS, Deng RM, Ma C (2021). Acyl-CoA synthetase long chain family member 4 plays detrimental role in early brain injury after subarachnoid hemorrhage in rats by inducing ferroptosis. CNS Neurosci Ther.

[134] Li YC, Wang R, Xu MM, Jing XR, Sun RB (2019). Aneurysmal subarachnoid hemorrhage onset alters pyruvate metabolism in poor-grade patients and clinical outcome depends on more: a cerebrospinal fluid metabolomic study. ACS Chem Neurosci.

[135] Dixon SJ, Patel DN, Welsch M, Skouta R, Lee ED, Hayano M (2014). Pharmacological inhibition of cystine-glutamate exchange induces endoplasmic reticulum stress and ferroptosis. Elife.

[136] Suzuki H, Kanamaru H, Kawakita F, Asada R, Fujimoto M, Shiba M (2021). Cerebrovascular pathophysiology of delayed cerebral ischemia after aneurysmal subarachnoid hemorrhage. Histol Histopathol.

[137] Bell JD, Thomas TC, Lass E, Ai J, Wan H, Lifshitz J (2014). Platelet-mediated changes to neuronal glutamate receptor expression at sites of microthrombosis following experimental subarachnoid hemorrhage. J Neurosurg.

[138] Helbok R, Kofler M, Schiefecker AJ, Gaasch M, Rass V, Pfausler B (2017). Clinical use of cerebral microdialysis in patients with aneurysmal subarachnoid hemorrhage-state of the art. Front Neurol.

[139] Lv H, Zhen C, Liu J, Yang P, Hu L, Shang P (2019). Unraveling the potential role of glutathione in multiple forms of cell death in cancer therapy. Oxid Med Cell Longev.

[140] Yang WS, SriRamaratnam R, Welsch ME, Shimada K, Skouta R, Viswanathan VS (2014). Regulation of ferroptotic cancer cell death by GPX4. Cell.

[141] Sato H, Tamba M, Kuriyama-Matsumura K, Okuno S, Bannai S (2000). Molecular cloning and expression of human xCT, the light chain of amino acid transport system xc. Antioxid Redox Signal.

[142] Ottestad-Hansen S, Hu QX, Follin-Arbelet VV, Bentea E, Sato H, Massie A (2018). The cystine-glutamate exchanger (xCT, Slc7a11) is expressed in significant concentrations in a subpopulation of astrocytes in the mouse brain. Glia.

[143] Brigelius-Flohe R, Maiorino M (2013). Glutathione peroxidases. Biochim Biophys Acta.

[144] Hao S, Liang B, Huang Q, Dong S, Wu Z, He W (2018). Metabolic networks in ferroptosis. Oncol Lett.

[145] Bersuker K, Hendricks JM, Li Z, Magtanong L, Ford B, Tang PH (2019). The CoQ oxidoreductase FSP1 acts parallel to GPX4 to inhibit ferroptosis. Nature.

[146] Shimada K, Hayano M, Pagano NC, Stockwell BR (2016). Cell-line selectivity improves the predictive power of pharmacogenomic analyses and helps identify NADPH as biomarker for ferroptosis sensitivity. Cell Chem Biol.

[147] Gao SQ, Liu JQ, Han YL, Deji QZ, Zhaba WD, Deng HJ (2020). Neuroprotective role of glutathione peroxidase 4 in experimental subarachnoid hemorrhage models. Life Sci.

[148] Jiao D, Xu J, Lou C, Luo Y, Ni C, Shen G (2023). Quercetin alleviates subarachnoid hemorrhage-induced early brain injury via inhibiting ferroptosis in the rat model. Anat Rec (Hoboken).

[149] Doll S, Freitas FP, Shah R, Aldrovandi M, da Silva MC, Ingold I (2019). FSP1 is a glutathione-independent ferroptosis suppressor. Nature.

[150] Yuan B, Zhao XD, Shen JD, Chen SJ, Huang HY, Zhou XM (2022). Activation of SIRT1 alleviates ferroptosis in the early brain injury after subarachnoid hemorrhage. Oxid Med Cell Longev.

[151] Zolnourian A, Galea I, Bulters D (2019). Neuroprotective role of the Nrf2 pathway in subarachnoid haemorrhage and its therapeutic potential. Oxid Med Cell Longev.

[152] Wang Z, Chen G, Zhu WW, Zhou D (2010). Activation of nuclear factor-erythroid 2-related factor 2 (Nrf2) in the basilar artery after subarachnoid hemorrhage in rats. Ann Clin Lab Sci.

[153] Li T, Wang H, Ding Y, Zhou M, Zhou X, Zhang X (2014). Genetic elimination of Nrf2 aggravates secondary complications except for vasospasm after experimental subarachnoid hemorrhage in mice. Brain Res.

[154] Chen J, Wang Y, Li M, Zhu X, Liu Z, Chen Q, et al. Netrin-1 alleviates early brain injury by regulating ferroptosis via the PPARγ/Nrf2/GPX4 signaling pathway following subarachnoid hemorrhage. Transl Stroke Res. 2023;15(1):219–37. 10.1007/s12975-022-01122-4 36631632

[155] Ma SJ, Li C, Yan C, Liu N, Jiang GY, Yang HR (2023). Melatonin alleviates early brain injury by inhibiting the NRF2-mediated ferroptosis pathway after subarachnoid hemorrhage. Free Radic Biol Med.

[156] Bollong MJ, Lee G, Coukos JS, Yun H, Zambaldo C, Chang JW (2018). A metabolite-derived protein modification integrates glycolysis with KEAP1-NRF2 signalling. Nature.

[157] Chen G, Fang Q, Zhang J, Zhou D, Wang Z (2011). Role of the Nrf2-ARE pathway in early brain injury after experimental subarachnoid hemorrhage. J Neurosci Res.

[158] Zhang T, Wu P, Budbazar E, Zhu Q, Sun C, Mo J (2019). Mitophagy reduces oxidative stress via Keap1 (Kelch-like epichlorohydrin-associated protein 1)/Nrf2 (nuclear Factor-E2-related factor 2)/PHB2 (prohibitin 2) pathway after subarachnoid hemorrhage in rats. Stroke.

[159] Schallner N, Pandit R, LeBlanc R, Thomas AJ, Ogilvy CS, Zuckerbraun BS (2015). Microglia regulate blood clearance in subarachnoid hemorrhage by heme oxygenase-1. J Clin Invest.

[160] Fan H, Ding R, Liu W, Zhang X, Li R, Wei B (2021). Heat shock protein 22 modulates NRF1/TFAM-dependent mitochondrial biogenesis and DRP1-sparked mitochondrial apoptosis through AMPK-PGC1alpha signaling pathway to alleviate the early brain injury of subarachnoid hemorrhage in rats. Redox Biol.

[161] Theil EC (2011). Ferritin protein nanocages use ion channels, catalytic sites, and nucleation channels to manage iron/oxygen chemistry. Curr Opin Chem Biol.

[162] Kurz T, Gustafsson B, Brunk UT (2006). Intralysosomal iron chelation protects against oxidative stress-induced cellular damage. FEBS J.

[163] Goodwin JM, Dowdle WE, DeJesus R, Wang Z, Bergman P, Kobylarz M (2017). Autophagy-independent lysosomal targeting regulated by ULK1/2-FIP200 and ATG9. Cell Rep.

[164] Hou W, Xie Y, Song X, Sun X, Lotze MT, Zeh HJ (2016). Autophagy promotes ferroptosis by degradation of ferritin. Autophagy.

[165] Gao M, Monian P, Pan Q, Zhang W, Xiang J, Jiang X (2016). Ferroptosis is an autophagic cell death process. Cell Res.

[166] Bellelli R, Federico G, Matte’ A, Colecchia D, Iolascon A, Chiariello M (2016). NCOA4 deficiency impairs systemic iron homeostasis. Cell Rep.

[167] Dowdle WE, Nyfeler B, Nagel J, Elling RA, Liu S, Triantafellow E (2014). Selective VPS34 inhibitor blocks autophagy and uncovers a role for NCOA4 in ferritin degradation and iron homeostasis in vivo. Nat Cell Biol.

[168] Mancias JD, PontanoVaites L, Nissim S, Biancur DE, Kim AJ, Wang X (2015). Ferritinophagy via NCOA4 is required for erythropoiesis and is regulated by iron dependent HERC2-mediated proteolysis. Elife.

[169] Wu H, Niu H, Wu C, Li Y, Wang K, Zhang J (2016). The autophagy-lysosomal system in subarachnoid haemorrhage. J Cell Mol Med.

[170] Jing CH, Wang L, Liu PP, Wu C, Ruan D, Chen G (2012). Autophagy activation is associated with neuroprotection against apoptosis via a mitochondrial pathway in a rat model of subarachnoid hemorrhage. Neuroscience.

[171] Liang Y, Deng Y, Zhao J, Liu L, Wang J, Chen P (2022). Ferritinophagy is involved in experimental subarachnoid hemorrhage-induced neuronal ferroptosis. Neurochem Res.

[172] Yu Y, Xie Y, Cao L, Yang L, Yang M, Lotze MT (2015). The ferroptosis inducer erastin enhances sensitivity of acute myeloid leukemia cells to chemotherapeutic agents. Mol Cell Oncol.

[173] Sun X, Ou Z, Xie M, Kang R, Fan Y, Niu X (2015). HSPB1 as a novel regulator of ferroptotic cancer cell death. Oncogene.

[174] Yuan H, Li X, Zhang X, Kang R, Tang D (2016). CISD1 inhibits ferroptosis by protection against mitochondrial lipid peroxidation. Biochem Biophys Res Commun.

[175] Kuang H, Wang T, Liu L, Tang C, Li T, Liu M (2021). Treatment of early brain injury after subarachnoid hemorrhage in the rat model by inhibiting p53-induced ferroptosis. Neurosci Lett.

[176] Zhang XS, Wu Q, Wu LY, Ye ZN, Jiang TW, Li W (2016). Sirtuin 1 activation protects against early brain injury after experimental subarachnoid hemorrhage in rats. Cell Death Dis.

[177] Zhang J, Zhu Q, Peng Z, Li XJ, Ding PF, Gao S, et al. Menaquinone-4 attenuates ferroptosis by upregulating DHODH through activation of SIRT1 after subarachnoid hemorrhage. Free Radic Biol Med. 2023;210:416–29. 10.1016/j.freeradbiomed.2023.11.031 38042225

[178] Codazzi F, Pelizzoni I, Zacchetti D, Grohovaz F (2015). Iron entry in neurons and astrocytes: a link with synaptic activity. Front Mol Neurosci.

[179] Ishii T, Warabi E, Mann GE (2019). Circadian control of BDNF-mediated Nrf2 activation in astrocytes protects dopaminergic neurons from ferroptosis. Free Radic Biol Med.

[180] Liu Y, Wang Z, Cao C, Xu Z, Lu J, Shen H (2022). Aquaporin 4 depolarization-enhanced transferrin infiltration leads to neuronal ferroptosis after subarachnoid hemorrhage in mice. Oxid Med Cell Longev.

[181] Domingues HS, Portugal CC, Socodato R, Relvas JB (2016). Oligodendrocyte, astrocyte, and microglia crosstalk in myelin development, damage, and repair. Front Cell Dev Biol.

[182] Xiao D, Qu Y, Pan L, Li X, Mu D (2018). MicroRNAs participate in the regulation of oligodendrocytes development in white matter injury. Rev Neurosci.

[183] Li Y, Wang B, Yang J, Liu R, Xie J, Wang J (2023). Iron overload causes ferroptosis but not apoptosis in MO3.13 oligodendrocytes. Neurochem Res.

[184] Shi J, Xue X, Yuan L, He G, Jiang Z, Wang L (2023). Amelioration of white matter injury through mitigating ferroptosis following hepcidin treatment after spinal cord injury. Mol Neurobiol.

[185] Weiner GM, Ozpinar A, Ducruet AF (2016). The role of matrix metalloproteinase-9 in subarachnoid hemorrhage-induced white matter injury. Neurosurgery.

[186] Shen D, Wu W, Liu J, Lan T, Xiao Z, Gai K (2022). Ferroptosis in oligodendrocyte progenitor cells mediates white matter injury after hemorrhagic stroke. Cell Death Dis.

[187] Fu W, Che X, Tan J, Cui S, Ma Y, Xu D, et al. Rasd1 is involved in white matter injury through neuron-oligodendrocyte communication after subarachnoid hemorrhage. CNS Neurosci Ther. 2023:e14452. 10.1111/cns.14452 PMC10916428 37735980

[188] Kettenmann H, Hanisch UK, Noda M, Verkhratsky A (2011). Physiology of microglia. Physiol Rev.

[189] Sabri M, Kawashima A, Ai J, Macdonald RL (2008). Neuronal and astrocytic apoptosis after subarachnoid hemorrhage: a possible cause for poor prognosis. Brain Res.

[190] Sun Y, Chen P, Zhai B, Zhang M, Xiang Y, Fang J (2020). The emerging role of ferroptosis in inflammation. Biomed Pharmacother.

[191] Tobin MK, Bonds JA, Minshall RD, Pelligrino DA, Testai FD, Lazarov O (2014). Neurogenesis and inflammation after ischemic stroke: what is known and where we go from here. J Cereb Blood Flow Metab.

[192] Qu W, Cheng Y, Peng W, Wu Y, Rui T, Luo C (2022). Targeting iNOS alleviates early brain injury after experimental subarachnoid hemorrhage via promoting ferroptosis of M1 microglia and reducing neuroinflammation. Mol Neurobiol.

[193] Kooijman E, Nijboer CH, van Velthoven CT, Mol W, Dijkhuizen RM, Kesecioglu J (2014). Long-term functional consequences and ongoing cerebral inflammation after subarachnoid hemorrhage in the rat. PLoS One.

[194] Cao Y, Li Y, He C, Yan F, Li JR, Xu HZ (2021). Selective ferroptosis inhibitor liproxstatin-1 attenuates neurological deficits and neuroinflammation after subarachnoid hemorrhage. Neurosci Bull.

[195] Heinsberg LW, Weeks DE, Alexander SA, Minster RL, Sherwood PR, Poloyac SM (2021). Iron homeostasis pathway DNA methylation trajectories reveal a role for STEAP3 metalloreductase in patient outcomes after aneurysmal subarachnoid hemorrhage. Epigenetics Commun.

[196] Zheng B, Zhou X, Pang L, Che Y, Qi X (2021). Baicalin suppresses autophagy-dependent ferroptosis in early brain injury after subarachnoid hemorrhage. Bioengineered.

[197] Tao Q, Qiu X, Li C, Zhou J, Gu L, Zhang L (2022). S100A8 regulates autophagy-dependent ferroptosis in microglia after experimental subarachnoid hemorrhage. Exp Neurol.

[198] Cao C, Lu T, Cheng Q, Cui G, Wang Z, Li X (2023). Restoring system xc-activity by xCT overexpression inhibited neuronal ferroptosis and improved neurological deficits after experimental subarachnoid hemorrhage. Brain Res.

